# An Uncharacteristic Presentation of Evans Syndrome Following Treatment With Dupilumab

**DOI:** 10.7759/cureus.16658

**Published:** 2021-07-27

**Authors:** Megan Rutherford, Minh Tran, Leonardo Salazar, Fatima Iqbal, Anam Mazharuddin, Julieanna Camarena

**Affiliations:** 1 Department of Internal Medicine, University of Texas Medical Branch, Galveston, USA; 2 Department of Pathology and Laboratory Medicine, University of Texas Medical Branch, Galveston, USA; 3 Department of Ophthalmology and Visual Sciences, University of Texas Medical Branch, Galveston, USA

**Keywords:** evans syndrome, autoimmune hemolytic anemia, thrombocytopenia, roth spots, dupilumab, blurry vision

## Abstract

Evans syndrome is a rare autoimmune disorder where patients develop autoimmune hemolytic anemia (AIHA), immune thrombocytopenia (ITP), and less commonly immune neutropenia. Patients typically present with fatigue, pallor, jaundice, petechiae, or epistaxis. A 27-year-old man with a history of atopic dermatitis for which he recently began treatment with dupilumab presented to the emergency department with a headache and blurry vision. Multiple Roth spots were seen on fundoscopic examination. Laboratory studies were consistent with warm AIHA, confirmed by a positive direct antiglobulin test (DAT), and severe thrombocytopenia. He was diagnosed with Evans syndrome. He was treated with corticosteroids, rituximab, and intravenous immunoglobulin (IVIG). His recovery was prolonged with the slow improvement of anemia and thrombocytopenia. This is an atypical presentation of Evans syndrome with isolated symptoms of new-onset blurry vision and headache along with the finding of Roth spots. Another interesting feature in the case is the recent use of dupilumab. Dupilumab is a monoclonal antibody that inhibits the T-helper cells type 2 (Th2) signaling pathway by blocking interleukin (IL)-4 and IL-13 binding. This alteration in the immune response could have a role in the development of Evans syndrome.

## Introduction

Evans syndrome is a rare autoimmune disorder that affects two or more blood cell lineages [1]. As a result of the disease, patients develop autoimmune hemolytic anemia (AIHA), immune thrombocytopenia (ITP), and less frequently immune neutropenia [1]. It typically presents with pallor, fatigue, jaundice, petechiae, or mucosal bleeding [1]. Diagnosis involves clinical history and characteristic laboratory studies. Laboratory studies in Evans syndrome include anemia, thrombocytopenia, reticulocytosis, unconjugated hyperbilirubinemia, elevated lactate dehydrogenase (LDH), low haptoglobin, and direct antiglobulin test (DAT) positive for warm immunoglobulin G (IgG) antibodies [1,2]. After the diagnosis of Evans syndrome is made, it can be further classified as primary or secondary Evans syndrome. Clinicians can diagnose primary Evans syndrome after other underlying conditions are excluded [1]. Secondary Evans syndrome is associated with many disorders including several autoimmune diseases, congenital or acquired immunodeficiencies (e.g., common variable immunodeficiency, immunoglobulin A (IgA) deficiency), infections, and malignancies [1]. Evans syndrome is difficult to manage as it can have slow or partial improvement with frequent exacerbations [1]. First-line treatments include corticosteroids and intravenous immunoglobulins (IVIG) [1,2]. Second-line treatments consist of rituximab, mycophenolate mofetil, cyclosporine, and splenectomy [1,2].

## Case presentation

A 27-year-old man presented to the emergency department with a two-week history of throbbing occipital headache and a two-day history of blurry vision with floaters. He had a history of atopic dermatitis and began treatment with dupilumab two months prior dosed at 200 mg every two weeks. He received his most recent dose of dupilumab one week before the presentation.

His physical examination was only notable for mild tachycardia. An outside ophthalmology clinic performed a dilated eye examination the day before that revealed multiple flame hemorrhages near the optic nerve, macular edema, cotton wool spots, and multiple Roth spots. High-quality retinal images were not obtained at the time. An underlying hematologic condition was suspected and the patient was admitted to the hospital. As infective endocarditis was on the differential diagnosis due to the presence of Roth spots, a set of blood cultures and a transthoracic echocardiogram were ordered. The results were unremarkable.

**Figure 1:**
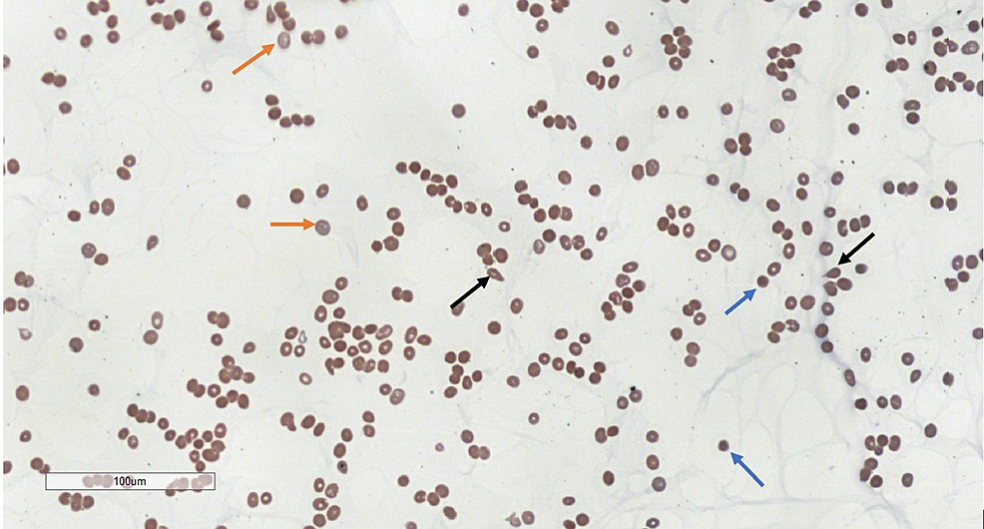
Peripheral blood smear showing anisopoikilocytosis (black arrows pointing to teardrop cells), spherocytes (blue arrows), polychromasia of red blood cells (red arrows), and extremely decreased platelets.

The initial complete blood count was significant for a white cell count of 5.64 × 10^3^/L, hemoglobin of 3.5 g/µdL with the mean corpuscular volume of 127.5, hematocrit of 10.2%, and platelet count of <2 × 10^3^/µL. Additional laboratory studies included total bilirubin of 5.5 mg/dL with unconjugated bilirubin of 4.7 mg/dL, creatinine of 0.81 mg/dL, LDH of 996 U/L, haptoglobin of <6 mg/dL, and reticulocyte percentage of 9.76%. He had unremarkable iron studies, folate level, vitamin B12 level, liver enzymes, fibrinogen, D-dimer, and ADAMTS13 activity. Based on these laboratory studies, AIHA was suspected and confirmed with a DAT positive for warm IgG antibody and low complement component 3 (C3) level. Figure 1 is an image from the peripheral blood smear, which revealed anemia with teardrop cells, spherocytes, leukocytes with granulocytic left shift, reactive lymphopenia, and severe thrombocytopenia.

**Figure 2:**
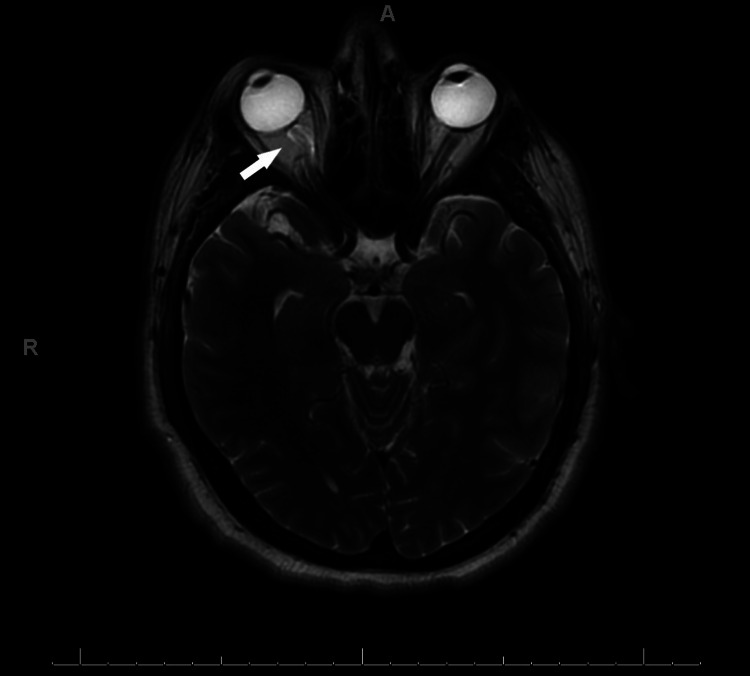
An MRI of the eye, orbit, and brain that is notable for a tortuous optic nerve sheath complex on the right (white arrow).

On admission, a computerized tomography (CT) scan of the head was completed to rule out an intracranial cause of his acute onset of headache. There were no intracranial abnormalities or mass effects noted on the CT. The CT thorax and abdomen/pelvis showed mildly enlarged axillary and mediastinal lymph nodes, hepatosplenomegaly, and a few subcentimeter retroperitoneal, iliac, and inguinal lymph nodes. Magnetic resonance imaging (MRI) of the eye, orbit, and brain was ordered. The MRI revealed a tortuous optic nerve sheath complex on the right side with no retrobulbar or intracranial mass, as seen in Figure 2.

**Figure 3:**
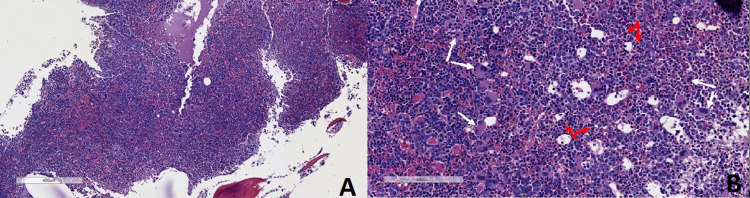
Bone marrow biopsy. (A) Low power view showing hypercellularity (cell to fat ratio 85-90%); (B) high power view showing increased megakaryocytes (white arrows) and erythroid precursors (red arrows).

**Figure 4:**
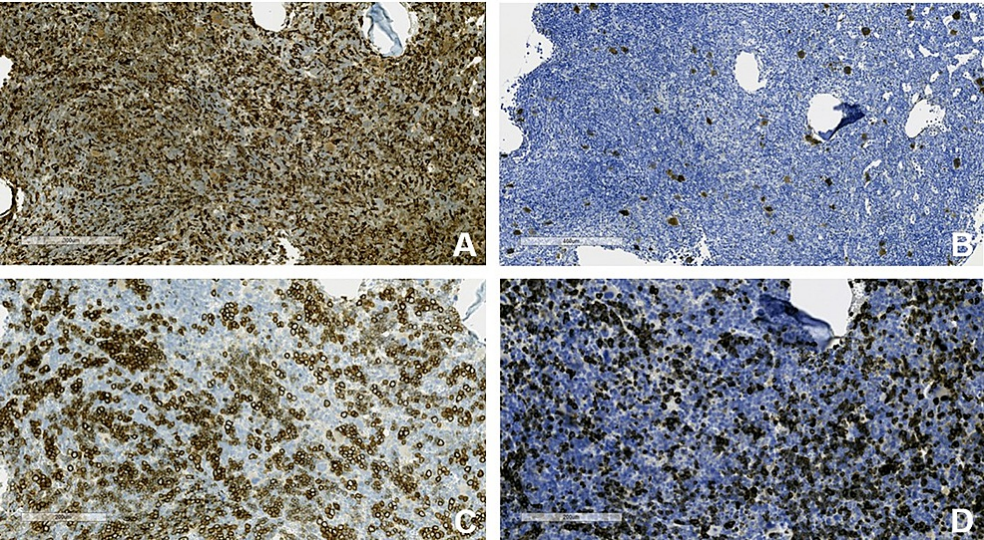
Immunostains of the bone marrow. (A) CD68 immunostain highlighting increased monocytes; (B) CD61 immunostain highlighting increased megakaryocytes; (C) E-Cadherin immunostain highlighting increased erythroid precursors; (D) myeloperoxidase immunostain highlighting the myeloid precursors.

**Figure 5:**
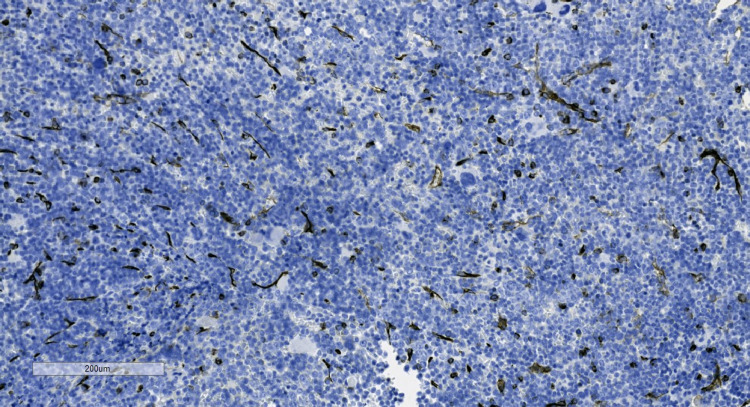
CD34 immunostain of the bone marrow highlighting blood vessels and few cells only. No increased or abnormal blast cells were seen.

The diagnosis was presumed to be Evans syndrome as he had AIHA and severe thrombocytopenia. To determine secondary causes of Evans syndrome, the patient underwent a bone marrow biopsy at the left posterior iliac crest and a full rheumatologic workup. Results of the bone marrow biopsy are shown in Figures 3A and 3B, 4A-4D, and 5. There was no evidence of lymphoproliferative disorder or acute leukemia by flow cytometry. Regarding the rheumatologic work-up, anti-nuclear antibody (ANA) was positive with a titer of 1:320 in a speckled pattern. However, other rheumatologic laboratory tests, including anti-SSA, anti-SSB, anti-double-stranded deoxyribonucleic acid (anti-dsDNA), anti-ribonucleoprotein (anti-RNP), and anti-Smith, were negative. After discussion with the patient, a lymph node biopsy was not performed due to his bleeding risk. The lymphadenopathy was followed with imaging.

**Figure 6:**
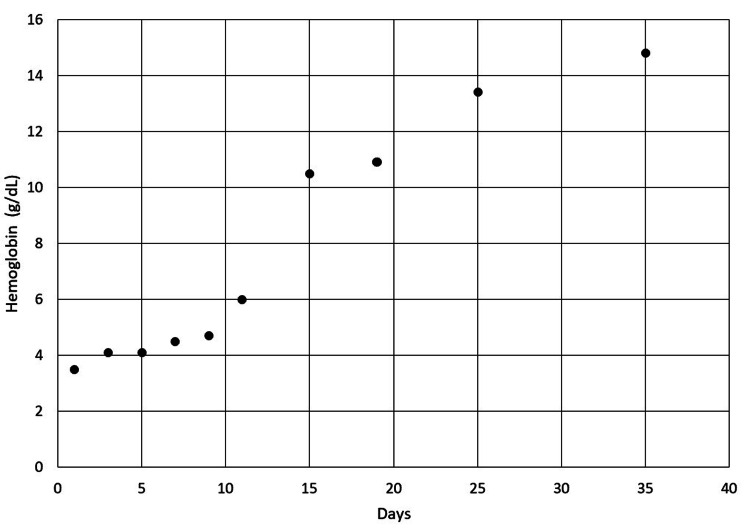
Improvement of the patient’s hemoglobin level in the days following treatment.

**Figure 7:**
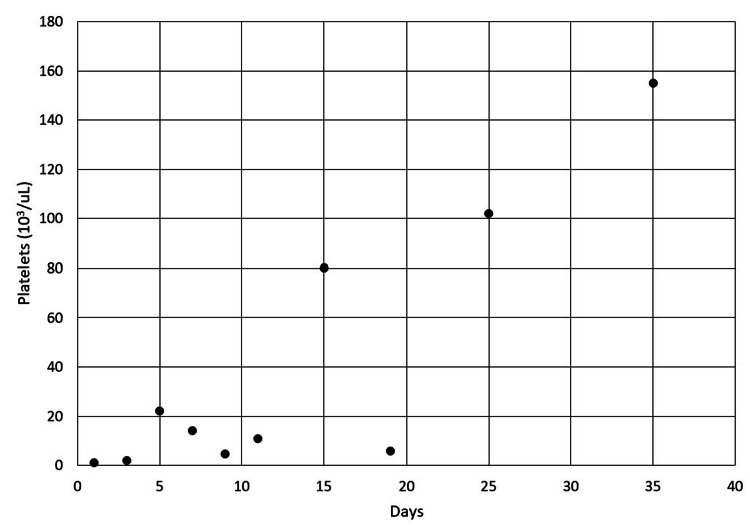
Improvement of the patient’s platelet count in the days following treatment.

He received treatment with glucocorticoids, IVIG, and several rounds of rituximab. During his first admission, he received IVIG twice, rituximab infusion at 375 mg/m^2^ three times a week, and methylprednisolone 150 mg twice a day for ten days followed by prednisone 50 mg daily for seven days. He left the hospital against medical advice on hospital day 9. Eleven days later, he had recurrent thrombocytopenia requiring readmission despite current treatment with prednisone 50 mg daily. He was given two additional rounds of treatment with rituximab and prednisone 120 mg daily with a slow taper over 60 days. After receiving treatment for more than one month, both his anemia and thrombocytopenia resolved as evident in Figures 6 and 7. His dilated fundus examination seven days into his first admission showed improvement in the size of his hemorrhagic pools following treatment. His blurry vision mildly improved, and he continued to follow with a retina specialist.

## Discussion

Evans syndrome is a rare autoimmune disorder where patients develop AIHA, ITP, and less commonly immune neutropenia. Our patient did not present with the classic symptoms of Evans syndrome. Instead, he had headaches, blurry vision, and the finding of Roth spots. Roth spots are white-centered retinal hemorrhages that can be caused by retinal capillary rupture and intraretinal bleeding [3]. They are commonly associated with infectious endocarditis but can be found in other conditions such as anemia, thrombocytopenia, leukemia, collagen vascular disease, hypertensive or diabetic retinopathy, and HIV infection [3]. There is one case report of a child with Evans syndrome who presented with right retinal hemorrhage and left premacular hemorrhage in addition to the loss of vision, gingival bleeding, and pallor [4].

Common autoimmune diseases associated with Evans syndrome are systemic lupus erythematosus, antiphospholipid syndrome, and Sjogren syndrome [1,2]. The diagnoses require rheumatologic laboratory tests for specific autoantibodies. Associated malignancies include non-Hodgkin lymphoma, chronic myelocytic leukemia, and chronic lymphocytic leukemia [1,2]. Bone marrow biopsy and flow cytometry are helpful in the workup of Evans syndrome to assess for malignancies [2]. For our patient, the workup for these possible secondary causes was only positive for ANA, which is nonspecific.

Some people are more susceptible to autoimmune diseases, and exposure to a specific trigger can result in them developing an autoimmune disease [5]. The use of vaccination and drugs have been suggested as potential triggers of secondary Evans syndrome [1,5,6]. There is one case report of Evans syndrome four days after the influenza vaccine and another case report two days after the hepatitis B vaccine [5,6]. Monoclonal antibodies have been implicated in AIHA including nivolumab [7], ipilimumab [7], alemtuzumab [8], natalizumab [9], efalizumab [10], adalimumab [11], and rituximab [12]. One patient out of 357 patients in a study on pancreas transplant recipients developed AIHA and ITP 22 months after beginning treatment with alemtuzumab and mycophenolate [13]. The mechanism is still unclear as these drugs have unique mechanisms of action despite being under the shared category of monoclonal antibodies.

Our patient was recently started on dupilumab for atopic dermatitis, and his last dose was one week before symptom onset. The recent use of dupilumab raises the question of its possible involvement in the development of Evans syndrome. Despite a relatively new appearance, dupilumab has been reported to have a safe profile with conjunctivitis and injection-site reaction noted as common side effects [14]. During the open-label extension study funded by Sanofi and Regeneron Pharmaceuticals, Inc. (Paris and Tarrytown, NY), 28 patients out of the 2,677 patients developed anemia and 8 patients developed thrombocytopenia [15]. One of the patients in the study developed a severe case of hemolytic anemia but further information was not provided [15]. There has also been one case report of a man who was diagnosed with acquired hemophilia A four weeks after he began treatment with dupilumab [16]. Dupilumab is a human monoclonal IgG antibody that inhibits the T-helper cells type 2 (Th2) signaling pathway induced by interleukin (IL)-13 and IL-4 at the IL-4 receptor, which decreases the inflammatory effect in asthma, atopic dermatitis, and other dermatologic conditions [14,17]. This decrease in Th2 signaling could cause a shift toward T-helper cell type 1 (Th1) and T-helper cell type 17 (Th17) activity [18]. Th1 cells are associated with autoimmune diseases, which could be a result of the increase in inflammatory cytokines [19]. This increase in Th1 activity was the proposed mechanism for developing cutaneous lupus in a patient after beginning treatment with dupilumab [18]. The continued use of dupilumab for atopic dermatitis requires a thorough risk and benefit consideration.

## Conclusions

This is the first case report to the best of our knowledge linking the recent use of dupilumab to an uncharacteristic presentation of Evans syndrome. Clinical suspicion should remain high for Evans syndrome in patients with thrombocytopenia and AIHA with negative work-up for malignancy and autoimmune diseases. Once Evans syndrome is diagnosed, it is important to assess for secondary causes. The role of dupilumab in this case of Evans syndrome is still unclear and further research is necessary to elucidate this association.
